# Scalable production and complete biophysical characterization of poly(ethylene glycol) surface conjugated liposome encapsulated hemoglobin (PEG-LEH)

**DOI:** 10.1371/journal.pone.0269939

**Published:** 2022-07-08

**Authors:** Uddyalok Banerjee, Savannah Wolfe, Quintin O’Boyle, Clayton Cuddington, Andre F. Palmer

**Affiliations:** William G. Lowrie Department of Chemical and Biomolecular Engineering, College of Engineering, The Ohio State University, Columbus, Ohio, United States of America; Brandeis University, UNITED STATES

## Abstract

Particle encapsulated hemoglobin (Hb)-based oxygen (O_2_) carriers (HBOCs) have clear advantages over their acellular counterparts because of their larger molecular diameter and lack of vasoactivity upon transfusion. Poly(ethylene glycol) surface conjugated liposome encapsulated Hb (PEG-LEH) nanoparticles are considered a promising class of HBOC for use as a red blood cell (RBC) substitute. However, their widespread usage is limited by manufacturing processes which prevent material scale up. In this study, PEG-LEH nanoparticles were produced via a scalable and robust process using a high-pressure cell disruptor, and their biophysical properties were thoroughly characterized. Hb encapsulation, methemoglobin (metHb) level, O_2_-PEG-LEH equilibria, PEG-LEH gaseous (oxygen, carbon monoxide, nitric oxide) ligand binding/release kinetics, lipocrit, and long-term storage stability allowed us to examine their potential suitability and efficacy as an RBC replacement. Our results demonstrate that PEG-LEH nanoparticle suspensions manufactured via a high-pressure cell disruptor have Hb concentrations comparable to whole blood (~12 g/dL) and possess other desirable characteristics, which may permit their use as potential lifesaving O_2_ therapeutics.

## Introduction

Blood supply continuity is a critical prerequisite for transfusions, routine treatment of chronic hematologic diseases and surgeries in both inpatient and outpatient facilities [1]. Shortages in the blood supply chain [2–4], seasonal demand spikes [5–7], short shelf-life of stored red blood cells (RBCs) [8, 9], and the hypothermic storage lesion [9–11] compounded by the acute need for large volumes of transfusable blood products in emergency situations such as wars and pandemics motivate the need to develop artificial RBC substitutes that are universally compatible [12, 13], pathogen free and are just as safe and efficacious as native RBCs.

Over the last few decades, liposome encapsulated hemoglobin (LEH) particles have gained considerable attention as RBC substitutes [14–18]. These cellular hemoglobin (Hb)-based oxygen (O_2_) carriers (HBOCs) encapsulate Hb inside their aqueous core, and can mitigate typical side-effects associated with earlier generations of commercially developed acellular HBOCs [14, 15]. Apart from their structural resemblance to natural RBCs, their large particle diameter (~250 nm) [19, 20] prevents particle extravasation into the tissue space through pores lining the blood vessel walls (fenestration diameter ~ 100nm) [21], and prevents subsequent interference with nitric oxide (NO) homeostasis generated via the endothelial cell layer [19]. In contrast, smaller-sized acellular HBOCs (diameters ~ 5–11 nm) [22, 23] can escape into the tissue space to readily scavenge NO, and elicit vasoconstriction at the microcirculatory level, systemic hypertension, and oxidative tissue toxicity [24–27]. Additionally, the vesicle membrane provides structural stability and compartmentalizes the encapsulated Hb to prevent the cytotoxic effects elicited by cell-free Hb in the circulation [19]. Typically, cell-free Hb in the circulation binds to the scavenger protein haptoglobin (Hp), and the resulting Hb-Hp complexes are cleared from the circulation by macrophages in the liver and spleen [28, 29]. However, this natural Hb clearance mechanism can be overwhelmed in the presence of excess cell-free Hb such as in sickle cell anemia, malaria or hemorrhagic shock, resulting in deleterious Hb filtration through the kidneys (i.e. hemoglobinuria) and oxidative renal injury [19, 27]. Encapsulating Hb within vesicles can completely eliminate the possibility of Hb excretion through the kidneys [19]. Moreover, safe removal of these phospholipid vesicles is achieved by phagocytosis via the reticuloendothelial system (RES) [30, 31].

**Fig 1:**
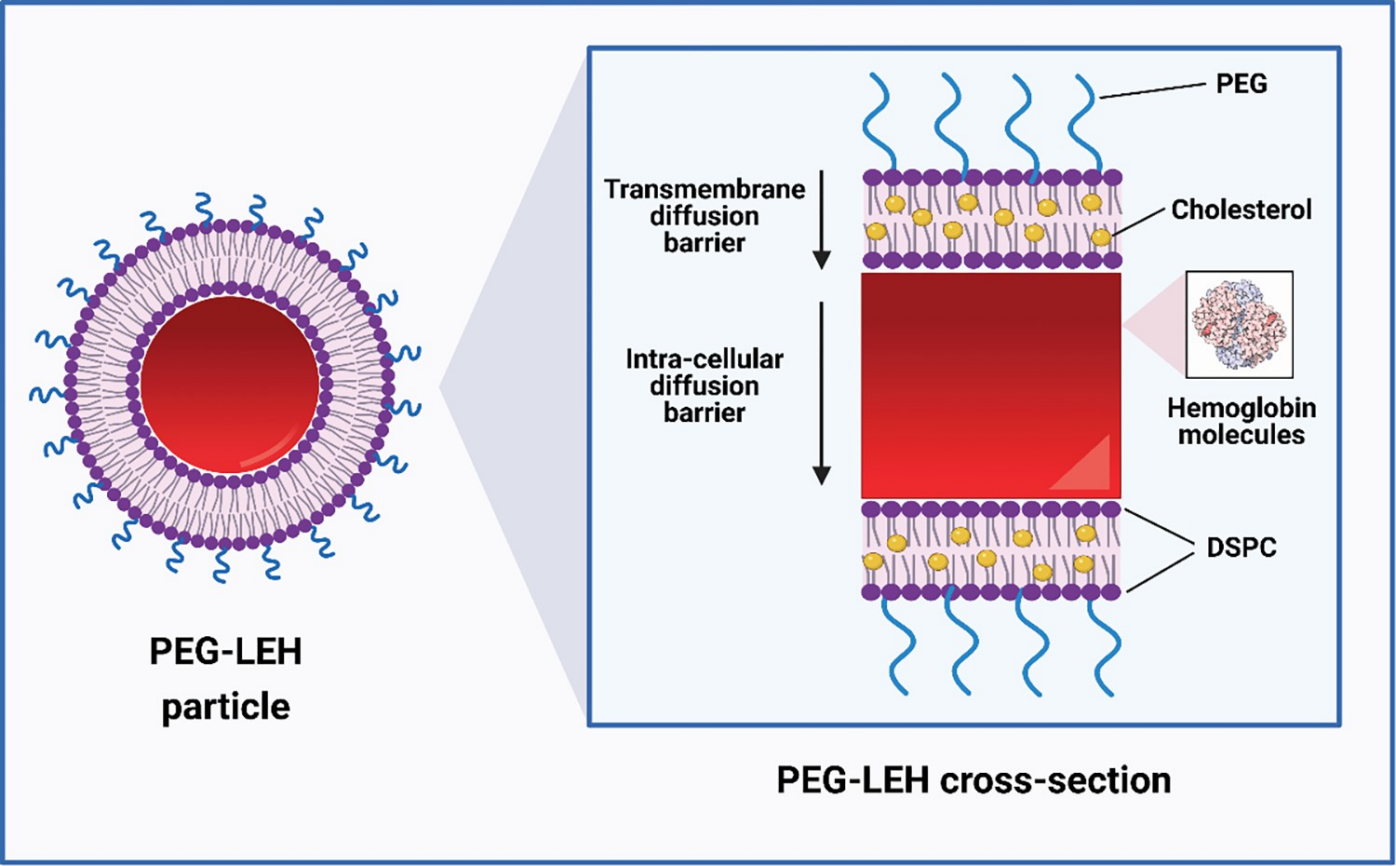
Structure of PEG-LEH nanoparticle. Inset: cross-section of a PEG-LEH nanoparticle with a more detailed breakdown of the structure and potential gaseous diffusion barriers [104]. Figure created with BioRender.com.

In earlier studies involving LEHs, it was observed that these materials possessed short circulatory half-lives, and were prone to aggregation after several days in storage [32]. It has been shown that these issues can be avoided by surface conjugating LEHs with poly(ethylene glycol) (PEG), a U.S. Food and Drug Administration (FDA) approved hydrophilic polymer used extensively in drug delivery systems [32, 33]. PEG surface conjugated LEHs (PEG-LEHs) provide numerous advantages over RBCs, such as the absence of blood-borne pathogens and blood group antigens, longer shelf-life, and higher resistance to shear stress and oxidative damage [15, 32, 33]. Furthermore, PEG-LEHs demonstrate improved stability *in vivo* compared to liposomes prepared with other common lipids [34, 35]. A schematic representation of the PEG-LEH structure is presented in **Fig 1**.

Most techniques for preparing PEG-LEH nanoparticles described in the literature are complex, time-consuming, non-scalable, and expensive [32, 33, 36]. Techniques using detergent dialysis and reverse phase evaporation can prove harmful to the structural stability and activity of Hb, and may even denature or chemically modify the encapsulated Hb [37]. Synthesis of large LEH particles (diameters ~ 2,400 nm) can lead to capillary blockage, which may in turn cause infarctions upon administration *in vivo* [36, 38]. The use of membrane extruders in the LEH synthesis process to control particle size and achieve unilamellar vesicles makes process scale-up difficult due to issues such as the high rate of membrane fouling and low permeation rates of the lipid/Hb suspension through the extruder membrane [39]. Moreover, some studies have purified Hb for encapsulation using dichloromethane [40] while others have heat pasteurized Hb at 60°C [36, 41]. The use of organic solvents and heat can potentially denature Hb. Kure and Sakai reported high encapsulation efficiency (~74% yield) Hb vesicles (Hb-V) using a kneading process in a 2-axes rotation-revolution mixer [41]. However, these Hb-Vs (and many others before) [33, 36, 42, 43] use 1,2-dipalmitoyl-*sn*-glycero-3-phosphatidylcholine (DPPC) as one of the key components to construct the lipid bilayer. DPPC has a phase-transition temperature of 41°C, which is very close to the core body temperature (37°C). Therefore, DPPC vesicles may exhibit increased membrane fluidity *in vivo* and can potentially disintegrate leading to the release of toxic free Hb into the blood stream, especially in the presence of comorbidities such as hyperpyrexia.

In order to mitigate these challenges, our group has developed an economical and innovative process [15] to synthesize PEG-LEH nanoparticles for use as RBC substitutes. The procedures constituting our formulation technique are individually scalable and can produce high throughput HBOCs which are physiologically stable, of ideal size for sustained circulation [20] and exhibit RBC-like biophysical properties. The current study expands upon the work of Rameez and Palmer [15], who engineered stable and homogenous PEG-LEH nanoparticles using a physiologically viable lipid recipe, ultrapure Hb (concentration > 30 g/dL), and a robust production methodology using a high-pressure membrane extruder. In this study, we scaled up the PEG-LEH nanoparticle production process using a high-pressure cell disruptor and consistently synthesized PEG-LEH nanoparticles at a Hb concentration comparable to whole blood (~ 12 g/dL). To assess the potential suitability and efficacy of these biomaterials as RBC substitutes, we performed complete *in vitro* biophysical characterization of these nanoparticles. The PEG-LEH nanoparticle size distribution, concentration of Hb in the suspension, metHb level inside the liposomes, O_2_-PEG-LEH equilibria, O_2_ off-loading rate constant, carbon monoxide (CO) binding rate constant, NO dioxygenation rate constant, lipocrit, internal Hb concentration inside the PEG-LEH nanoparticle, long term storage stability, and encapsulation of Hb bound to CO and NO were evaluated and reported. Overall, this work demonstrates large-scale production via a high-pressure cell disruptor and thorough biophysical characterization of PEG-LEH nanoparticles for use as a potentially safe and efficacious RBC substitute.

## Materials and methods

### Materials

Distearoyl-phosphatidylcholine (DSPC), poly(ethylene glycol)_5000_-distearoyl-phosphatidylethanolamine (PEG_5000_-DSPE), and cholesterol were used to synthesize PEG-LEH nanoparticles. DSPC was purchased from Avanti Polar Lipids (Alabaster, AL); while PEG_5000_-DSPE and cholesterol were procured from Laysan Bio Inc. (Arab, AL) and Sigma-Aldrich (St. Louis, MO) respectively. The liposome suspension was subjected to diafiltration on a 500 kDa hollow fiber tangential flow filtration (TFF) module (ID: M1-500S-360-01S) purchased from Spectrum Laboratories (Rancho Dominguez, CA). All other chemicals were obtained from Sigma-Aldrich (St. Louis, MO). Expired leuko-reduced human RBCs were procured from the American Red Cross (Columbus, OH).

### Purification of human Hb from RBCs

Human Hb (hHb) was purified from lysed human RBCs via TFF as described by Palmer et al. [44]. Hb concentration and metHb levels were determined using the Winterbourne equation as described in the literature [45].

### PEG-LEH synthesis

Prior to encapsulation inside the aqueous core of lipid vesicles, Hb was converted into its carbonyl (HbCO) form. Encapsulating Hb in the HbCO form ensures the oxidative stability of Hb during the LEH nanoparticle production process, as HbCO is approximately 200 times more stable than oxy-Hb (HbO_2_) against auto-oxidation [46]. Complete conversion of HbO_2_ to HbCO was achieved by taking 100 mL of concentrated HbO_2_ solution in a glass bottle and saturating the head space with CO gas (99.3%), in the absence of light, and under gentle stirring for 4–5 hours. The complete conversion of HbO_2_ to HbCO was confirmed via UV-visible spectroscopy.

PEG-LEH nanoparticle suspensions were prepared by the method of Rameez and Palmer with some key changes [15]. Briefly, a 1:1 molar ratio of DSPC and cholesterol (mass ratio: 3.6 g DSPC for 1.4 g cholesterol) was completely dissolved in chloroform by manual shaking. The chloroform was evaporated using a rotary evaporator to form a white lipid film on the inner surface of the container. The lipid film was then vacuum-dried for an additional 48 hours followed by hydration with 100 mL of HbCO ([Hb] > 350 mg/mL) suspended in phosphate buffered saline (PBS, 0.1 M, pH 7.4) in a 1 L round bottom flask. The lipid/Hb solution was thoroughly mixed at 25°C for 14 h in a CO saturated atmosphere to form multi-lamellar liposome encapsulated Hb (LEH) particles. The multi-lamellar LEH suspension was then passed through a cell disruptor (Constant Systems, Daventry, UK) 2–3 times in batch mode with a pressure-head of 10,000 psi to form unilamellar LEH nanoparticles. This process for converting the multilamellar LEH suspension into unilamellar LEH nanoparticles, differs from the membrane extruder process utilized by Rameez and Palmer [15]. After each pass through the high-pressure cell disruptor, the average particle diameter was measured using dynamic light scattering (DLS). Once the particle diameter reached < 300 nm, homogenization was followed by outer surface PEGylation of the LEH nanoparticles. PEG_5000_-DSPE (1% w/v) suspended in PBS was added to the homogenized LEH/Hb nanoparticle suspension. A 1:2 (v/v) ratio of PEG solution to LEH/Hb nanoparticle suspension was maintained to keep the PEG concentration below its critical micelle concentration (CMC) [47]. Insertion of PEG_5000_-DSPE into the outer membrane of LEH nanoparticles was allowed to proceed at 55–60°C for 1 h under a CO saturated atmosphere. The nanoparticle suspension was gently stirred to promote insertion of the PEG-lipid into the outer membrane of the LEH nanoparticle.

The PEG-LEH nanoparticle suspension was diafiltered at 25°C with PBS over a 500 kDa TFF cartridge to remove unencapsulated Hb and free lipids. The washed PEG-LEH nanoparticle suspension was further concentrated using a 500 kDa TFF cartridge (Spectrum Labs, Rancho Dominguez, CA).

### PEG-LEH nanoparticle size distribution

An Eclipse^®^ asymmetric flow field-flow fractionator (A4F) (Wyatt Technology Corp., Santa Barbara, CA) connected in series to an 18-angle Dawn Heleos^®^ multi-angle static light scattering (MASLS) photometer (Wyatt Technology Corp., Santa Barbara, CA) was used to measure the absolute size distribution of PEG-LEH nanoparticles as described in the literature [48, 49]. The MASLS photometer was equipped with a 30 mW GaAs laser operating at a wavelength of 658 nm. ASTRA 5.3 software (Wyatt Technology Corp., Santa Barbara, CA) was used to analyze the light scattering spectra and calculate the PEG-LEH nanoparticle absolute size distribution. PBS was used as the elution buffer. To further confirm the particle size, the average hydrodynamic diameter of PEG-LEH nanoparticle dispersions were measured at 37°C using a Zetasizer Nano DLS spectrometer (Malvern Instruments Ltd., Worcestershire, United Kingdom).

### Hb and MetHb concentration inside PEG-LEH nanoparticles

The concentration of Hb and metHb level inside PEG-LEH nanoparticles were measured after lysing the vesicles with Triton X-100 as described by Rameez and Palmer [14, 50]. Briefly, 100 μL of PEG-LEH nanoparticle suspension was diluted with 800 μL PBS. The diluted nanoparticle suspension was then heated to 5–10°C above the phase transition temperature of DSPC (55°C) for 20–25 minutes. 100 μL 10% v/v Triton X-100 was added to the heated vesicle suspension and mixed thoroughly for 1–2 minutes. Immediately afterwards, the suspension was centrifuged at 20,000g for 5–10 minutes. Post-centrifugation, the supernatant was collected and concentrations of released Hb and metHb were measured using UV-visible spectroscopy and the Winterbourne equation [45].

### O_2_-PEG-LEH equilibria measurements

HbCO encapsulated inside PEG-LEH nanoparticles was photolyzed via irradiation with visible light in an O_2_ saturated atmosphere. Briefly, 2–3 mL of concentrated PEG-LEH nanoparticle suspension was diluted with PBS in a 1:2 (v/v) ratio and placed in a sealed serum bottle. The nanoparticle suspension was degassed, and photolysis was then carried out in the presence of visible light (GE Edison SP 10° 90 W light bulb) in an O_2_ saturated atmosphere for 15–20 minutes. The serum bottle was placed in an ice-bath to maintain the temperature at 4°C throughout the process in order to minimize metHb formation. The conversion of HbCO to HbO_2_ was confirmed by measuring the absorption spectra of Hb derived from lysed PEG-LEH nanoparticle suspensions via UV-visible spectroscopy [45]. The O_2_-PEG-LEH equilibrium binding curves were generated using a Hemox Analyzer (TCS Scientific Corp., New Hope, PA) at 37°C (physiological temperature). Hb O_2_-saturation was plotted as function of the partial pressure of O_2_ (pO_2_) to yield the O_2_ equilibrium curve (OEC). The Hill equation was used to fit the OECs obtained for PEG-LEH nanoparticles and Hb [48]. The P_50_, or the pO_2_ at which 50% of the Hb is saturated with O_2_, and the cooperativity coefficient (n) of PEG-LEH nanoparticles were regressed from the Hill curve fit to the OEC and compared to corresponding values obtained for cell-free Hb and RBCs.

### Rapid kinetic measurements

PEG-LEH nanoparticle gaseous ligand binding/release kinetics was measured using an Applied Photophysics SF-17 microvolume stopped-flow spectrophotometer (Applied Photophysics Ltd., Surrey, United Kingdom) [14, 15, 51]. For all stopped flow measurements, a control of Hb was used to ensure the authenticity of results.

To measure the binding/release kinetics of O_2_-PEG-LEH nanoparticles, oxygenated Hb/PEG-LEH nanoparticle suspensions having an overall heme concentration of 15 μM were rapidly mixed with a 1.5 mg/mL sodium dithionite solution (Sigma-Aldrich, St. Louis, MO) in PBS. The O_2_ off-loading kinetics were monitored via measuring the absorbance at 437.5 nm and 20°C. Deoxygenation time courses of oxygenated PEG-LEH nanoparticles were recorded using the spectrophotometer pro-data software (SX17MV). O_2_ off-loading rate constants (k_off_, _O2_) were calculated as the slope of the linear plot of ln (absorbance) vs time (in seconds).

The CO association kinetics were similarly measured, and the reaction was also monitored at 437.5 nm and 20°C. CO-binding to deoxy-Hb is a second order reaction. To simplify analysis, a pseudo first order approximation was made. The reaction was carried out separately using two very high concentrations of CO (232 and 464 μM) as compared to the heme concentration (15 μM). The two apparent first order reaction rate constants obtained from these measurements were then plotted against the corresponding CO concentrations, and the slope of the linear fit yielded the second order rate constant for CO association (k_on, CO_).

The NO dioxygenation reaction involves the conversion of ferrous oxy-Hb to ferric Hb. This is a very fast reaction (reaction rates ~ 10^7^ M^-1^s^-1^), thus certain precautions were taken to effectively monitor this reaction. A very dilute solution of oxygenated PEG-LEH nanoparticles (1 μM on heme basis) was reacted with low concentrations of NO stock solution (12.5 and 25 μM). NO dioxygenation of the control (Hb) was monitored for the shortest time scale the instrument could measure (0.0125 s). For PEG-LEH nanoparticles, the lipid membrane and the intra-cellular diffusion barriers considerably lengthen the dioxygenation reaction; therefore, a longer time scale (1–2 s) was used to monitor their kinetics. The reaction was monitored at 420 nm and 20°C. Like CO association, NO dioxygenation is also a second order reaction. The NO stock solution was prepared by bubbling NO gas through a deoxygenated solution of 0.1 M phosphate buffer (PB), pH 7.4 as outlined by Rameez and Palmer [15]. The dioxygenation rate constant (k_ox, NO_) was determined in a manner similar to that of k_on, CO_ as described above.

### Lipocrit of PEG-LEH nanoparticle dispersions

PEG-LEH nanoparticle dispersions were diluted 10× in PBS and were ultra-centrifuged (L90K, Beckman Coulter Inc., Brea, CA) at 100,000g for 1 h. The lipocrit, or the percentage of packed PEG-LEH nanoparticles in the entire volume of solution, was then computed using the following equation:

$$Lipocrit=\frac{V_{1}-V_{2}}{V_{1}}*D*100$$ (1)

Where *V*_*1*_ is initial volume of PEG-LEH nanoparticle suspension, *V*_*2*_ is final supernatant volume after ultracentrifugation and *D* is the dilution factor. The lipocrit was used to calculate the number of PEG-LEH nanoparticles per mL of suspension (*N*) as shown below:

$$N=\frac{\left(\Phi *Lipocrit\right)}{V_{PEG-LEH}}$$ (2)

where *Φ* is packing fraction and *V*_*PEG-LEH*_ is the volume of an individual PEG-LEH nanoparticle which was computed from the PEG-LEH nanoparticle diameter obtained from DLS or asymmetric flow field-flow fractionation coupled with MASLS (A4F-MASLS). The number of Hb molecules encapsulated per PEG-LEH nanoparticle was computed by multiplying moles of Hb contained inside a PEG-LEH particle and Avogadro’s number. Internal [Hb] and molar mass of Hb (64,500 g/mol) were used to determine moles of Hb.


$$\begin{array}{c} No.of\;Hb\;molecules\;per\;PEG-LEH\;particle=\\ (moles\;of\;Hb\;inside\;a\;PEG-LEH\;particle)*Avogadro\prime s\;Number \end{array}$$ (3)

### Long term storage stability of PEG-LEH nanoparticles

PEG-LEH nanoparticles, stored at 4°C for 30 days, were diluted 10× in PBS and ultra-centrifuged (L90K, Beckman Coulter Inc., Brea, CA) at 100,000 g for 1 h. The supernatant was carefully removed, and its volume was recorded. The supernatant was assayed for total cell-free Hb released via UV-visible spectroscopy and analyzed using the Winterbourne equation [45]. The packed PEG-LEH nanoparticles were then re-suspended in 1 mL PBS and the total volume of the re-suspended vesicles was recorded. The Hb concentration inside the re-suspended PEG-LEH nanoparticles were obtained by lysing the vesicles with Triton X-100 using techniques described above. The total degree of lysis in the PEG-LEH nanoparticle suspension over the storage period was estimated using the following equations (Eqs 4–6):

$$\%\;Lysis=(\frac{mass\;of\;Hb\;in\;supernatant}{mass\;of\;Hb\;in\;supernatant+mass\;of\;Hb\;inside\;PEG-LEHs\;})*100$$ (4)

Where,

$$mass\;of\;Hb\;in\;supernatant={\left[Hb\right]}_{supernatant}*(volume\;of\;supernatant)$$ (5)

and,

$$mass\;of\;Hb\;inside\;PEG-LEH\;particles={\left[Hb\right]}_{PEG-LEH}\;*(volume\;of\;resuspended\;vesicles)$$ (6)

### PEG-LEH nanoparticles encapsulating HbCO and HbNO

During the preparation of PEG-LEH nanoparticles, it was necessary to convert the Hb into the HbCO form in order to ensure its oxidative stability (low metHb level) during processing [46]. Post PEG-LEH nanoparticle synthesis, the CO bound state of encapsulated Hb was confirmed via UV-visible spectroscopy after lysing the PEG-LEH nanoparticles to yield cell-free Hb using techniques described above. Since the affinity of Hb for NO is ~1000-fold greater than its affinity for CO [52], we attempted a direct displacement reaction to form HbNO encapsulated PEG-LEH nanoparticles by treating PEG-LEH nanoparticles encapsulating HbCO with ultra-pure NO gas. Deoxygenated PEG-LEH nanoparticles encapsulating HbCO were treated with 99.9% pure NO gas (bubbled through 5 M NaOH to remove nitrite impurities) for 3–6 h in a sealed environment and in the absence of light to convert the encapsulated Hb to its NO bound state. The NO bound state of Hb was then confirmed via UV-visible spectroscopy. The reaction scheme is as follows:

HbCO+NO→HbNO+CO (7)

Cell-free Hb was converted to its HbCO and HbNO forms to serve as controls in this experiment. Briefly, 5–10 mL concentrated Hb solution was taken in a sealed serum bottle and degassed for 20–30 min. The degassing process was continued until the solution pO_2_ dropped to 0 mm Hg and confirmed using a Blood Gas Analyzer (Siemens Rapidlab 248, Diamond Diagnostics, Holliston, MA). Ultra-pure (99.3%) CO gas was then passed through the serum bottle headspace for 1–2 h. The CO bound state of the Hb was confirmed via UV-visible spectroscopy. To form HbNO, 5–8 mL of concentrated HbCO solution was placed in a sealed serum bottle and treated with ultra-pure (99.9%) NO gas (bubbled through 5 M NaOH) for 1–2 h in the absence of light. All gassing and degassing experiments were carried out in an ice-bath at 4°C to limit metHb formation.

### Statistical analysis

Anova/2-sided *t-*tests were used to check statistical significance of differences in biophysical properties between Hb, RBCs and PEG-LEH nanoparticles. A p-value of p<0.05 was considered statistically significant. Statistical analyses were performed using JMP software (SAS Institute Inc., Cary, NC).

## Results and discussion

For this study, it was important to determine the size, Hb encapsulation, O2 carrying capacity, gaseous ligand binding/release kinetics, lipocrit, and long-term storage stability of PEG-LEH nanoparticles to judge their suitability as potential gaseous ligand carriers. Biophysical properties of hHb, RBCs, and PEG-LEH nanoparticle suspensions are listed in Table 1. For reference, biophysical properties reported by Rameez et al. are also contained in Table 1.

**Table 1:** Biophysical properties of PEG-LEH nanoparticles, RBCs and Hb.

Property	Human Hb (n = 12)	Human RBC (n = 12)	PEG-LEH (n = 12)	PEG-LEH [15] (Rameez) (n = 3)
Diameter (nm)	5.5 [57]	2000–8000 [51]	256 ± 20.81	176 ± 9.42
[Hb] in suspension (g/dL)	31.77 ± 8.70	22.51 ± 5.91	12.01 ± 1.29	11–13
MetHb (%)	0	0.35 ± 0.38	0.91 ± 0.1	<1.0%
k_off, O2_ (s^-1^)	36.51 ± 2.53	8.61 ± 2.55	11.18 ± 1.82	21.57
k_on, CO_ (μM^-1^s^-1^)	0.20 ± 0.01	0.09 ± 0.03	0.17 ± 0.01	0.212
k_ox, NO_ (μM^-1^s^-1^)	34.99 ± 7.46	0.33 ± 0.32	2.14 ± 0.76	4.00
P_50_ (mm Hg)	12.55 ± 0.97	27.16 ± 4.45	17.60 ± 1.72	22.87 ± 2.29
Cooperativity (n)	2.64 ± 0.09	2.21 ± 0.13	1.92 ± 0.12	2.11 ± 0.08
Lipocrit/Hematocrit (%)	-	70 ± 5	26.1 ± 2.7	20
Internal [Hb] (g/dL)	-	32.16 ± 8.44	46.3 ± 5.3	50–75
No. of PEG-LEH nanoparticles/mL dispersion (× 10^13^)	-	-	1.95 ± 0.44	NA
% Lysis after 30 days	-	> 1%	0.37 ± 0.30 *(n = 8)	0.71 ± 0.71 *(after 4–5 mon)
No. of molecules of gas transported by a PEG-LEH nanoparticle (× 10^3^)	-	-	154.46 ± 39.23	NA
No. of Hb molecules/PEG-LEH nanoparticle (× 10^3^)	-	-	38.62 ± 9.81	NA

### PEG-LEH nanoparticle size distribution

HBOC molecular diameter plays a critical role in determining the safety and efficacy of RBC substitutes [24, 25]. The small size of earlier generations of commercial acellular HBOCs is primarily responsible for the observed vascular side-effects when these HBOCs were administered *in vivo* [21]. HBOC size influences its ability to extravasate through the blood vessel wall and deposit in the tissue space. To avert these side-effects, researchers have developed HBOCs such as polymersome encapsulated Hb (PEH) nanoparticles and PEG-LEH nanoparticles, which are larger in size compared to the size of pores lining the inner walls of the vasculature [53–56].

**Fig 2:**
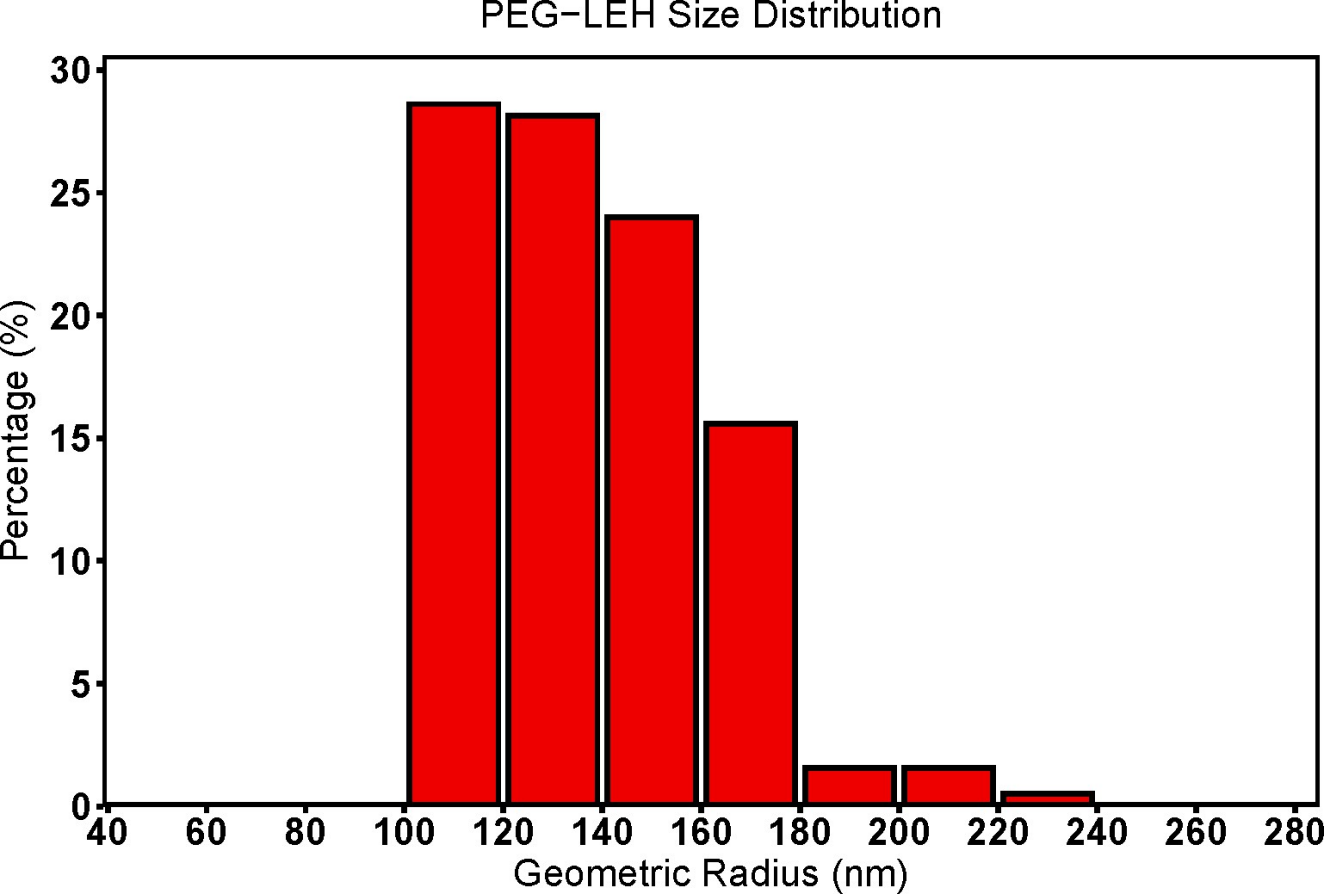
PEG-LEH nanoparticle size distribution. A typical PEG-LEH nanoparticle absolute size distribution plot generated using multi-angle static light scattering data obtained from a Dawn Heleos^®^ MASLS photometer. Nanoparticle fractionation was achieved using an Eclipse^®^ A4F system.

The average hydrodynamic diameter measured for the PEG-LEH nanoparticles synthesized in this study was 253 ± 27.25 nm via A4F-MASLS, and comparably 256 ± 20.81 nm via DLS. Additionally, the PEG-LEH nanoparticle size distribution was measured using A4F-MASLS. Fig 2 shows a typical PEG-LEH nanoparticle size distribution plot This data suggests that the nanoparticles produced in this study are monodisperse and should lead to minimal extravasation *in vivo*. This particle size range is slightly larger than the ideal nanoparticle size (~160–220 nm in diameter) for optimal circulation persistence [20, 36, 38]. Circulation persistence of vesicles in the blood stream varies inversely with particle diameter and directly with particle surface area to volume ratio [58–60]. In our PEG-LEH nanoparticle production method, the hydrodynamic diameter of these particles can be easily regulated by increasing the number of passes through the cell disruptor and/or using a higher pressure-head (15,000/20,000 psi instead of 10,000 psi) during the homogenization step. We were able to synthesize PEG-LEH nanoparticles having diameters ranging between 150–180 nm by increasing the number of passes and/or using a higher pressure-head. However, this adversely impacted Hb encapsulation within these vesicles ([Hb] ~ 6.5 g/dL). We believe that this loss in Hb encapsulation can be attributed to the rupture of vesicles upon exposure to additional shear forces resulting from the increased number of passes through the cell disruptor. We aimed to synthesize PEG-LEH nanoparticle dispersions with a solution concentration of Hb comparable to that of whole blood (15.7 g/dL for men and 13.8 g/dL for women) [61]. We deemed it necessary to strike a balance between nanoparticle size and Hb encapsulation. Hydrodynamic diameters observed for the liposomes produced in this study are significantly larger (p<0.05) than diameters reported for previous generations of acellular HBOCs (<100 nm) [22, 23, 62–66]. Therefore, these particles should potentially elicit minimum vasoactivity upon administration [21, 65].

### Hb and MetHb concentration inside PEG-LEH nanoparticles

Our goal was to produce PEG-LEH nanoparticle dispersions with a Hb concentration comparable to that of whole blood. Thus, it was critical to measure the concentration of Hb and % metHb of PEG-LEH nanoparticle dispersions. **Table 1** compares [Hb] observed in PEG-LEH nanoparticle suspensions, packed RBCs and cell-free Hb. We found that the [Hb] in solution was significantly higher in packed RBCs suspensions ([Hb] = 22.51 ± 5.91 g/dL) as compared to PEG-LEH nanoparticle suspensions ([Hb] = 12.01 ± 1.29 g/dL). However, the internal [Hb] was significantly higher inside PEG-LEH nanoparticles ([Hb] = 46.3 ± 5.3 g/dL) compared to inside RBCs ([Hb] = 32.16 ± 8.44 g/dL). Our observations make sense, given the relatively smaller diameters of these PEG-LEH nanoparticles.

**Table 1** also reports % metHb levels of the PEG-LEH nanoparticles synthesized in this study. Prior to liposome synthesis, the % metHb of the starting material (stock HbCO solution) was also measured by UV-visible spectroscopy and analyzed using the Winterbourne equation [45]. The metHb levels of stock HbCO solution were below detectable limits, whereas the metHb levels of the PEG-LEH dispersions were 0.91 ± 0.1%. The low metHb levels observed in the PEG-LEH nanoparticles can be attributed to the fact that all Hb was converted to its more stable HbCO form prior to encapsulation within the lipid vesicles [46]. To reduce auto-oxidation of encapsulated Hb, reducing agents such as ascorbic acid can be co-encapsulated along with Hb inside the vesicle’s aqueous core. Recent studies have highlighted the role of ascorbic acid/ascorbate in suppressing Hb auto-oxidation *in vivo* [9, 11, 66, 67].

In this study we synthesized ~20 mL batches of PEG-LEHs with ~46g/dL [Hb]. Our hHb encapsulation efficiencies ranged between 24–30% which is comparable to efficiencies reported in other extrusion based studies [19, 33, 36, 41]. However, these Hb encapsulation efficiencies are a fraction of the high yields (~74%) reported by Kure and Sakai [41]. The impressive yields observed in the kneading study have been attributed to better mixing of the highly viscous Hb–lipid mixture under elevated shear stress and reactor temperature (~60°C for 10 min kneading) [41]. Demanding reactor conditions can result in better yields, however, may also explain the high metHb levels (5–10%) observed in those Hb-Vs immediately after production. In contrast, the metHb levels observed within PEG-LEHs synthesized in this study were negligible (~1%). We find the trade-off with modest encapsulation efficiencies acceptable, as our process allows capturing unencapsulated Hb (from the diafiltration stages) and concentrating the same for future formulations.

### O_2_-PEG-LEH nanoparticle equilibria measurements

**Fig 3:**
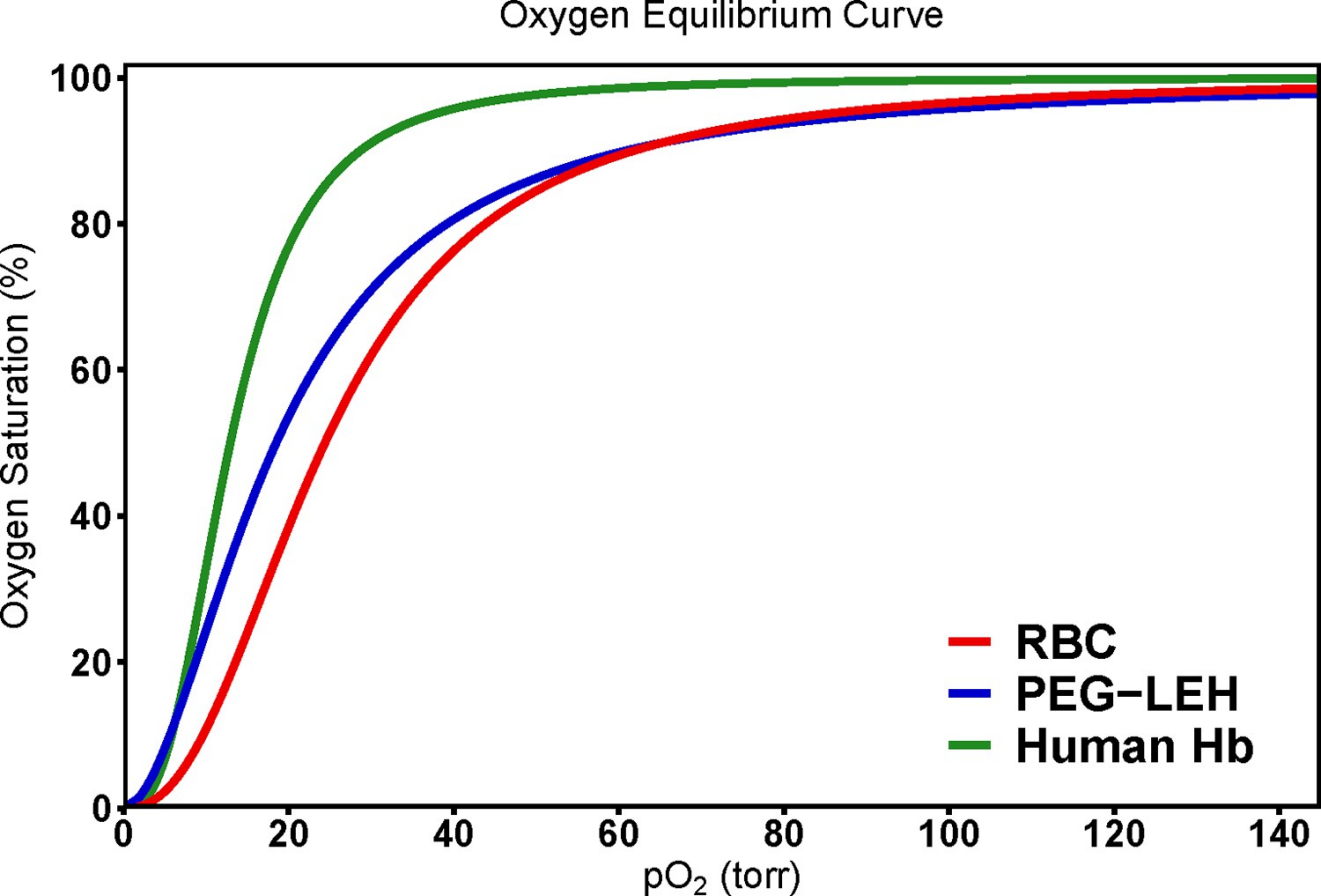
Comparison of O_2_ equilibrium curves (OECs) for RBCs, Human Hb and PEG-LEH nanoparticles.

O_2_-PEG-LEH nanoparticle equilibrium data were fit to the Hill equation to determine the P_50_ and cooperativity coefficient (n). **Table 1** lists the P_50_ and n of PEG-LEH nanoparticles prepared in this study. **Fig 3** compares typical OECs of RBCs, cell-free Hb and PEG-LEH nanoparticles. PEG-LEH nanoparticle OEC shapes were sigmoidal and similar to those obtained for RBCs and cell-free Hb. This indicates that the cooperative binding of O_2_ to Hb was not compromised by the PEG-LEH nanoparticle manufacturing process. P_50_ for PEG-LEH nanoparticles was higher (p<0.05) compared to cell-free hHb, and n was lower (p<0.05). These differences have been attributed to encapsulation of highly concentrated Hb solution within lipid vesicles [15]. ‘Crowding’ of encapsulated Hb molecules within PEG-LEHs is thought to inhibit quaternary conformational changes in proximal globin chains [15] observed in cell-free Hb during its transition from the deoxy, T-state to oxy R-state [68]. Both the P_50_ and n were lower (p<0.05) for PEG-LEH nanoparticles compared to RBCs. This observation is expected given that allosteric effectors were not co-encapsulated inside the PEG-LEH nanoparticles to control the P_50_ of the encapsulated Hb, with the P_50_ being consistent with values reported in the literature [34, 54, 69].

Cabrales et al. has previously shown that in ischemic tissues, where the pO_2_ < 10 mm Hg, phospholipid vesicles with low P_50_ (*~* 8 mm Hg) were the primary source of O_2_ being transported to tissues surpassing O_2_ bound to RBCs [70]. Additionally, the small size of these vesicles (~ 250 nm) allows them to perfuse through partially blocked capillaries which otherwise would obstruct the passage of much larger sized RBCs (~8,000 nm). Moreover, local pO_2_ levels in ischemic tissues are greatly reduced as blood flow through these vessels is extremely slow. RBCs with higher P_50_ (lower oxygen affinity) release most of their O_2_ in transit, before reaching the targeted ischemic tissues [71–73]. Therefore, the PEG-LEH nanoparticles produced in this study with moderate P_50_ values should be better-suited towards re-perfusing ischemic tissues. Additionally, under normoxic microcirculatory conditions, lower P_50_ values (compared to RBCs) of PEG-LEH nanoparticles will facilitate controlled O_2_ transfer from these HBOCs to tissues [15, 74].

### O_2_ rapid kinetic measurements

**Fig 4:**
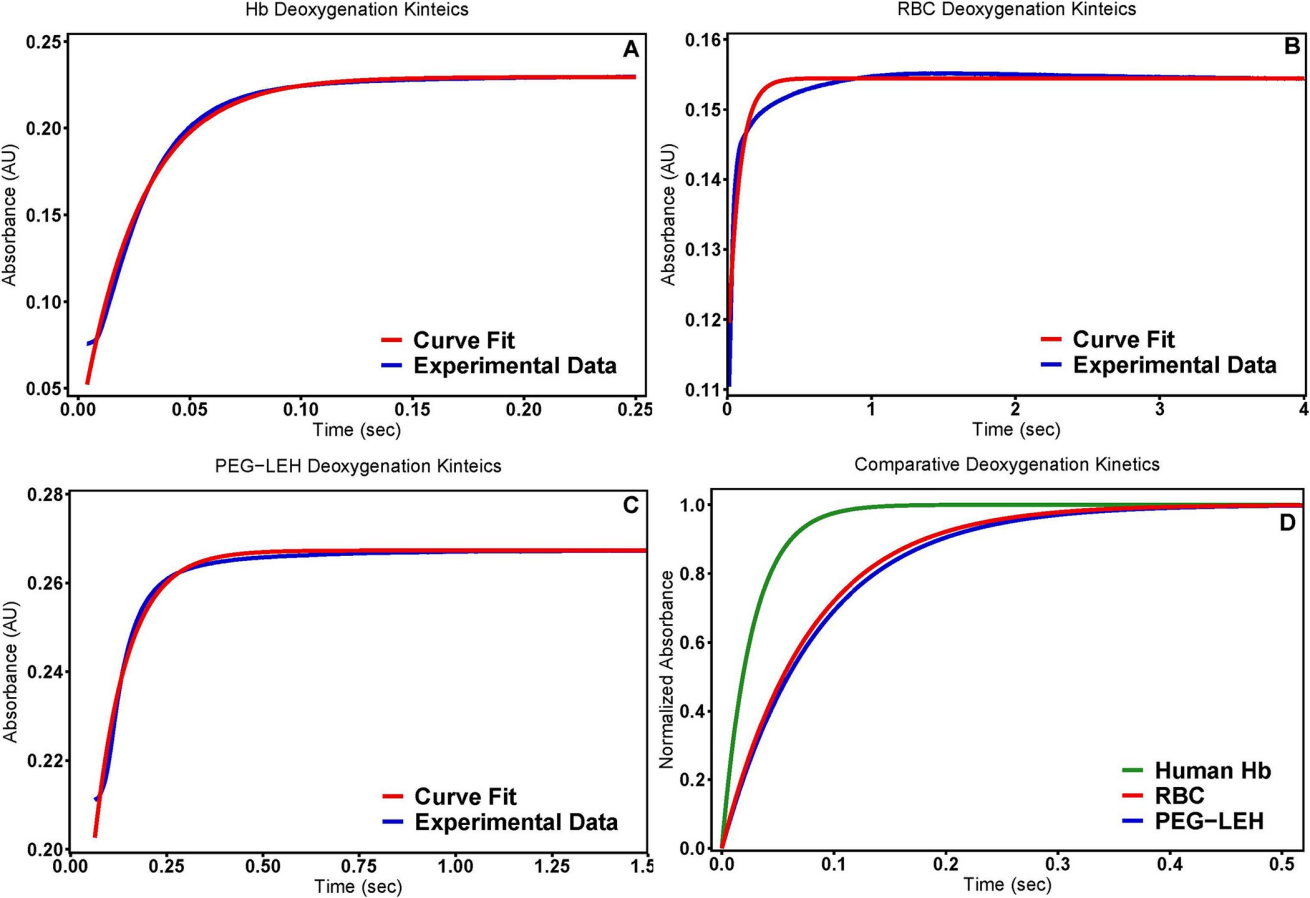
Deoxygenation kinetics. Time courses for deoxygenation of human (A) Hb, (B) RBCs, and (C) PEG-LEH nanoparticles were collected in the presence of 1.5 mg/mL sodium dithionite. The experimental data shows an average of 10–15 kinetic traces. D) The normalized absorbance of the experimental fit for the three groups is shown as a direct comparison of the deoxygenation kinetics. The reactions were monitored at 437.5 nm and 20°C. PBS (0.1 M, pH 7.4) was used as the reaction buffer.

The $k_{off,O_{2}}\;$ values (**Table 1**) obtained for the PEG-LEH nanoparticles ranged between 11.18 ± 1.82 s^-1^ and are slightly higher (p<0.05) than the values obtained for RBCs (8.61 ± 2.55 s^-1^). However, the $k_{off,O_{2}}$ rate constant for both PEG-LEH nanoparticles and RBCs are significantly lower (p<0.05) than the values obtained for the Hb control (34–41 s^-1^). **Fig 4** compares typical kinetic time courses of O_2_ dissociation for PEG-LEH nanoparticles, RBCs and cell-free Hb measured using an Applied Photophysics SF-17 microvolume stopped-flow spectrophotometer.

The $k_{off,O_{2}}$ rate constant obtained for PEG-LEH nanoparticles are 3-fold lower than the rate constant obtained for cell-free Hb. Comparable reduction in $k_{off,O_{2}}$ rate constant was observed for RBCs (4-fold) when compared to cell-free Hb. In general, similar reduction in O_2_ offloading rate constants have been previously reported in the literature for both PEG-LEH nanoparticles and RBCs [14, 15, 75]. Taken together, these observations support the fact that encapsulation of Hb inside the aqueous core of Hb carriers such as RBCs and PEG-LEH nanoparticles play a major role in controlling delivery of O_2_ to tissues. The $k_{off,O_{2}}$ rate constant for PEG-LEH nanoparticles produced in this study are slightly higher than the rate constant measured for RBCs. Intracellular diffusion barriers increase with particle size [69]. PEG-LEH nanoparticles are much smaller in size compared to RBCs, indicating that RBCs provide a larger intracellular diffusion barrier to O_2_ offloading.

High O_2_ offloading rates from Hb forms the basis of the ‘autoregulation theory’ for the development of vasoconstriction and systemic hypertension [76–78]. An increase in O_2_ delivery by HBOC facilitated diffusion, decreases O_2_ consumption by tissues due to vasoconstriction [21, 79]. Thus, moderate O_2_ release rates are critical in ascertaining HBOC efficacy. The PEG-LEH nanoparticles produced in the current study considerably retard O_2_ offloading as compared to acellular HBOCs [80] and cell-free Hb. Furthermore, their $k_{off,O_{2}}$ rate constant is comparable to that of RBCs. Thus, the PEG-LEH nanoparticles synthesized in this study can potentially deliver O_2_ to ischemic tissues at regulated rates avoiding vasoconstriction due to oversupply of O_2_.

### CO rapid kinetic measurements

**Fig 5:**
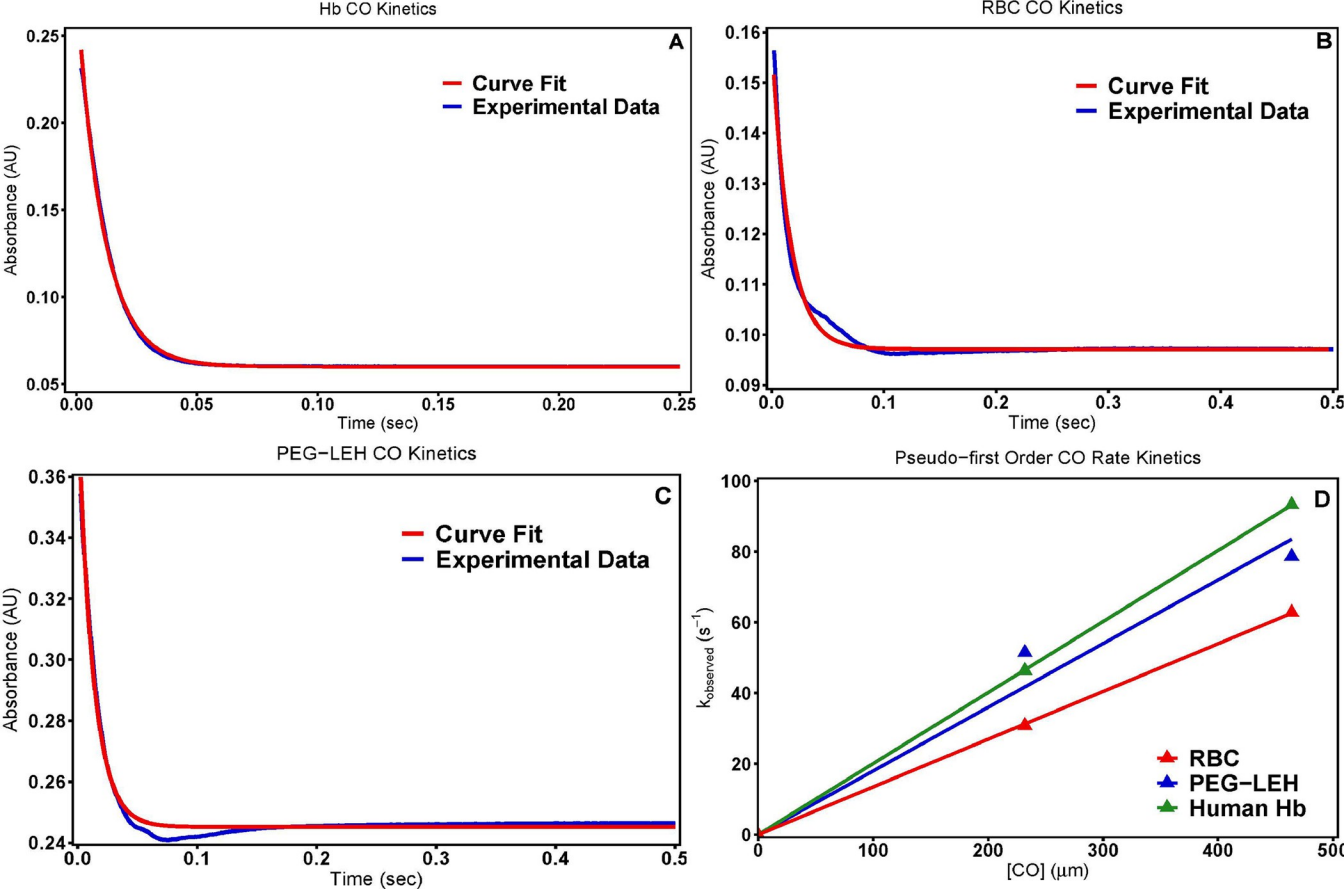
CO rate kinetics. Time courses for the CO (464 μM) association reaction with deoxygenated (A) Hb, (B) RBCs, and (C) PEG-LEH nanoparticles. The CO association reaction was carried out in presence of 1.5 mg/mL sodium dithionite. The experimental data shows an average of 10–15 kinetic traces. The reaction was monitored at 437.5 nm and 20°C. PBS was used as the reaction buffer. (D) Comparison of CO pseudo first order association rate constants for Hb, RBCs and PEG-LEH nanoparticles as a function of [CO].

**Fig 5** shows characteristic CO association kinetic time courses for deoxygenated human **(A)** Hb, **(B)** RBCs, and **(C)** PEG-LEH nanoparticles upon reaction with CO stock solution (464 μM). The time courses were similar for Hb, RBCs and PEG-LEH nanoparticles. **Fig 5D** plots the dependence of the pseudo-first order rate constants as a function of CO concentration for RBCs, Hb and PEG-LEH nanoparticles. Therefore, the slopes of the linear fits in **Fig 5D** yield the second order CO binding rate constants of the various species.

The *k*_*on*,*CO*_ values obtained for PEG-LEH nanoparticles ranged between 0.17 ± 0.01 μM^-1^s^-1^ (**Table 1**) and are significantly higher than the CO association rate constants obtained for RBCs (0.09 ± 0.03 μM^-1^s^-1^) (p<0.05). This observation can be explained by the larger intracellular diffusion barrier offered by RBCs owing to their much larger diameter as compared to PEG-LEH nanoparticles [81]. The *k*_*on*,*CO*_ rate constant obtained for cell-free Hb tested in this study ranged between 0.20 ± 0.01 μM^-1^s^-1^. These values are significantly higher (p<0.05) than the values obtained for RBCs. However, no significant difference was observed when these values were compared to the *k*_*on*,*CO*_ rates obtained for PEG-LEH nanoparticles. Therefore, we conclude that Hb encapsulation inside liposomes did not affect PEG-LEH nanoparticle CO association kinetics. Our observations are consistent with Sakai et al., who reported CO association rate constants of phospholipid vesicles using different PEG-LEH nanoparticle production techniques [81, 82]. It has been further noted that the association rate constants of these ligands follow their π electron accepting trends [83]. $k_{on,O_{2}}$ and *k*_*on*,*NO*_ rates for Hb reported in the literature 3.3 μM^-1^s^-1^ [84] and 25 μM^-1^s^-1^ [85, 86] suggest that subtle differences in the low magnitude CO binding rate constants were not adequately captured by the kinetic measurements performed in this study.

Dissociation rate constants of HbCO have been previously reported in the literature [52, 83]. We believe that analogous to CO association, PEG-LEH nanoparticles will release CO faster than RBCs due to their smaller intracellular diffusion barrier. CO is a gaseous autocrine/paracrine messenger which has many physiological roles in vasoprotection, and has long been investigated as a therapeutic for applications in vascular disease treatment, and hemorrhagic shock and resuscitation [87]. Prior *in vivo* studies using CO-releasing molecules as therapeutics demonstrated endothelial and neuroprotective effects [88–90]. Furthermore, CO bound chemically modified Hbs reduced myocardial infarct sizes in rats [91–93] and suppressed key pathways responsible for inducing vaso-occlusion crises in transgenic sickle mice models [92]. Other studies transfused cross-linked Hb tetramers ligated to CO to improve perfusion in ischemic cerebral arteries [94]. In light of these promising studies, we conclude that PEG-LEH nanoparticles should be able to potentially deliver exogenous CO to ischemic tissues at slow to moderate rates in order to circumvent reperfusion injury resulting from generation of reactive oxygen species (ROS) and oxidative tissue toxicity.

### NO rapid kinetic measurements

**Fig 6:**
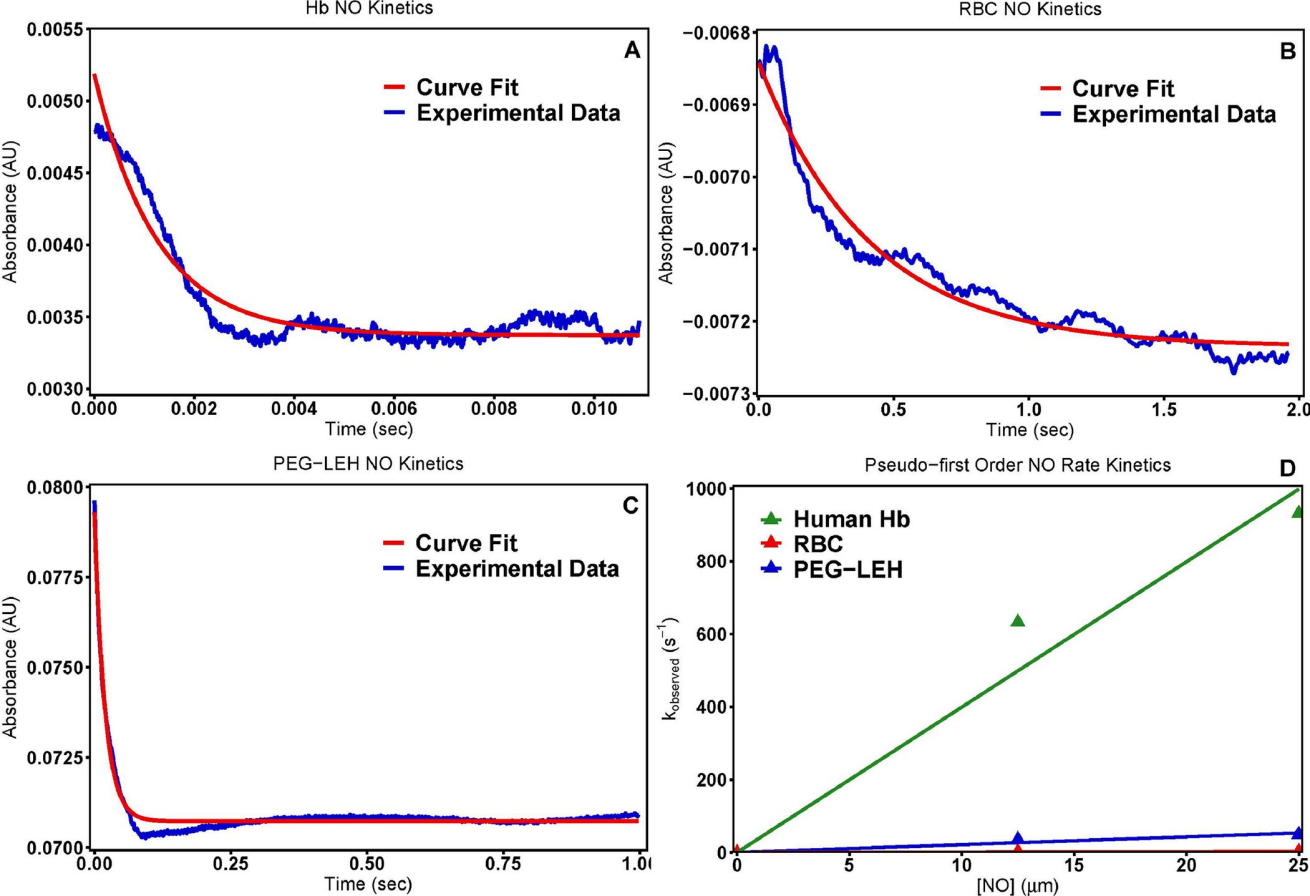
NO rate kinetics. Time courses for the NO (25 μM) dioxygenation reaction with oxygenated (A) Hb, (B) RBCs, and (C) PEG-LEH nanoparticles. The experimental data shows an average of 10–15 kinetic traces. Reactions were monitored at 420 nm and 20°C. (D) Comparison of pseudo first order NO dioxygenation rate constants for human Hb, RBCs and PEG-LEH nanoparticles as a function of [NO].

**Fig 6** shows characteristic NO dioxygenation kinetic time courses for oxygenated human **(A)** Hb, **(B)** RBCs, and **(C)** PEG-LEH nanoparticles upon reaction with NO stock solution (25 μM). **Fig 6D** shows the dependence of the observed pseudo first order rate constants on NO concentration for Hb, RBCs and PEG-LEH nanoparticles. Furthermore, the values for the NO dioxygenation rate constants (*k*_*ox*,*NO*_) obtained in this study are reported in **Table 1**. Low HbO_2_ concentrations (~ 0.5 μM on heme basis) used in these experiments led to subtle absorbance changes; therefore, an average of 10–15 kinetic traces were measured for each time course in order to enhance the signal-to-noise ratio.

The *k*_*ox*,*NO*_ values for both PEG-LEH nanoparticles and RBCs were significantly lower (p<0.05) than the values obtained for cell-free Hb (34.99 ± 7.46 μM^-1^s^-1^). As noted earlier, the intracellular diffusion barrier afforded by the large particle diameter and Hb shielding promotes this reduction in NO binding [81]. The *k*_*ox*,*NO*_ values of PEG-LEH nanoparticles synthesized in this study are 10–17 fold lower than the rate constants observed for cell-free Hb, 8–9 fold lower than prior generations of commercial acellular HBOCs [62, 63], and about 4 fold lower than the values reported for PEG-LEH nanoparticles produced using different methods [95]. NO dioxygenation rate constants obtained for PEG-LEH nanoparticles (2.14 ± 0.76 μM^-1^s^-1^) were significantly higher (p<0.05) than those obtained for RBCs (0.33 ± 0.32 μM^-1^s^-1^). As explained before, this observation is due to the smaller size of PEG-LEH nanoparticles leading to a smaller intracellular diffusion barrier as compared to RBCs. Interestingly, it has been shown via mathematical modelling that PEG-LEH nanoparticles exhibit comparable retardation to NO diffusion as RBCs when extrapolated to the size of RBCs [15, 95]. Taken together, the PEG-LEH nanoparticles synthesized in the current study should potentially induce very little NO scavenging mediated vasoconstriction and hypertension if administered *in vivo*.

### Summary of kinetic measurements

Monitoring the reaction kinetics of cell-free Hb, RBCs and PEG-LEH nanoparticles with physiologically relevant gaseous ligands (O_2_, CO and NO) were deemed important in order to test the viability of PEG-LEH nanoparticles as safe and efficacious RBC substitutes. The results from these rapid kinetic measurements showed that if administered *in vivo*, PEG-LEH nanoparticles can potentially deliver O_2_ to ischemic tissues at regulated rates, thereby averting vasoconstriction due to oversupply of O_2_. PEG-LEH nanoparticles could also potentially deliver CO to ischemic tissues at controlled rates in order to circumvent reperfusion injury resulting from ROS generation and oxidative tissue toxicity. Additionally, the low NO dioxygenation rate constants of these vesicles enable them to limit scavenging of endothelial NO, thus suppressing NO scavenging mediated vasoconstriction and hypertension. Therefore, the PEG-LEHs are ideal gaseous ligand delivery vessels because they interfere with little or none endogenously available CO or NO.

### Lipocrit of PEG-LEH dispersions

Lipocrit, the volume fraction of packed PEG-LEH nanoparticles in solution, is conceptually identical to the hematocrit, which is the volume fraction of whole blood that is occupied by packed RBCs [96]. Typical hematocrit levels observed range from 40–54% in men and 36–48% in women [97]. In comparison, the lipocrit for the PEG-LEH nanoparticles synthesized in this study was measured as 26.1 ± 2.7%. A typical calculation for estimating the lipocrit of these vesicles is shown below.


$$From\;Equation\;1,\;Lipocrit=\frac{V_{1}-V_{2}}{V_{1}}*D*100$$

$$∴Lipocrit=\frac{1000\;\mu L-975\;\mu L}{1000\;\mu L}*10*100=25\%$$

The lipocrit was used to calculate the number of PEG-LEH nanoparticles per mL of suspension (*N*) as shown below:

$$From\;Equation\;2,\;N=\frac{\left(\Phi *Lipocrit\right)}{V_{PEG-LEH}}$$

A value of *Φ* = 0.64 for random close packing of hard spheres was taken from the literature [98]. The diameter of a PEG-LEH nanoparticle (d_PEG-LEH_) measured via DLS was on average 256 nm.


$$V_{PEG-LEH}=volume\;of\;an\;individual\;PEG-LEH\;nanoparticle=\frac{4\pi }{3}{\left(\frac{d_{PEG-LEH}}{2}\right)}^{3}=8.8\times 10^{6}\;{nm}^{3}$$

Therefore,

$$N=\frac{\left(\Phi *Lipocrit\right)}{V_{PEG-LEH}}=\frac{0.64*\;0.25\;mL}{8.8*10^{-15}\;{cm}^{3}}=1.8\times 10^{13}\frac{particles}{mL}suspension$$

Using average internal [Hb] ~ 463.1 mg/mL and MW of Hb ~ 64.5 kDa,

Fromequation3,No.ofHbmoleculesperPEG−LEHnanoparticle=(molesofHbinsideaPEG−LEHnanoparticle)*Avogadro′sNumber

$$=\left(\frac{\left(8.8*\;10^{-15})*(463.1\right)}{64,500*10^{3}}\right)*[6.023*10^{23}]=38,055\;molecules$$

### Long term storage stability of PEG-LEH nanoparticles

For RBCs to be transfusable, the FDA mandates a total lysis of <1% during the 42-day *ex vivo* storage period [99, 100]. The total degree of lysis in PEG-LEH nanoparticles over their 30-day storage period was observed to be 0.37 ± 0.30%. Surface conjugation with PEG and storage of these vesicles in their CO form account for the low amount of Hb leakage observed over their storage period. Interestingly, no precipitation was observed when the PEG-LEH nanoparticles were stored at 4°C for extended periods of time. Surface modification of LEH nanoparticles with PEG is believed to prevent particle aggregation and is also responsible for the colloidal stability of these vesicles in storage [15, 32]. Nonetheless, a limitation of this study is that % lysis was not measured beyond 30 days, however, PEG-LEHs synthesized using similar methods have reported total lysis <1% after 4–5 months in storage (Table 1) [15]. In light of recent reports which evaluated storage stability of Hb-Vs for extended periods (1–2 years) [41, 43], we plan on conducting similar tests for PEG-LEHs in the future. Another interesting study will be to observe the effect on % lysis when reducing agents such as ascorbic acid is co-encapsulated.

### PEG-LEH nanoparticles encapsulating HbCO and HbNO

**Fig 7:**
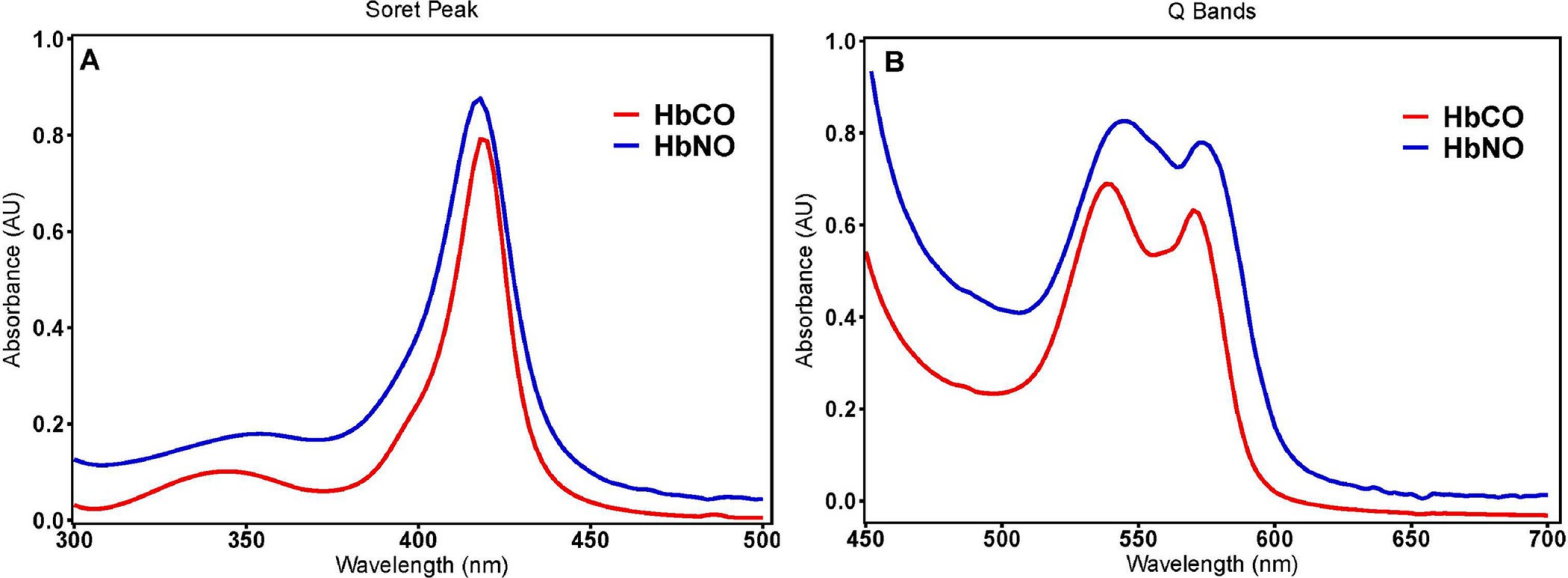
UV-visible spectroscopy of NO and CO bound cell free-Hb. (A) Soret and (B) Q Bands of NO and CO bound cell free-Hb measured were consistent with literature values, confirming PEG-LEH nanoparticle encapsulation of HbCO and HbNO.

Another objective of this study was to encapsulate CO and NO bound Hb inside the PEG-LEHs. This was deemed important as HBOCs delivering physiologically active gaseous ligands with proven anti-apoptotic, anti-inflammatory and vasorelaxing properties such as CO and NO can potentially mitigate reperfusion injuries encountered during ischemic shock resuscitation [46, 91–93, 101–103]. UV-visible spectroscopy was used to confirm HbCO and HbNO encapsulation within PEG-LEH nanoparticles. **Fig 7** compares the Soret band **(A)** and the Q bands **(B)** of CO (red) and NO (blue) bound cell free-Hb. The pure spectra of HbCO were obtained using UV-visible spectroscopy after treating cell-free Hb with ultra-pure CO gas. The HbCO spectra had a Soret peak at 419 nm (**Fig 7A**) and the Q bands were at 540 nm and 569 nm (**Fig 7B**). The pure spectra of HbNO were obtained using UV-visible spectroscopy following treatment of HbCO with ultra-pure NO gas. HbNO spectra had a Soret peak at 418 nm (**Fig 7A**) and the Q bands were at 545 nm and 575 nm (**Fig 7B**). Our observations are consistent with the UV-visible spectra of pure HbCO and HbNO reported in the literature [52].

**Fig 8:**
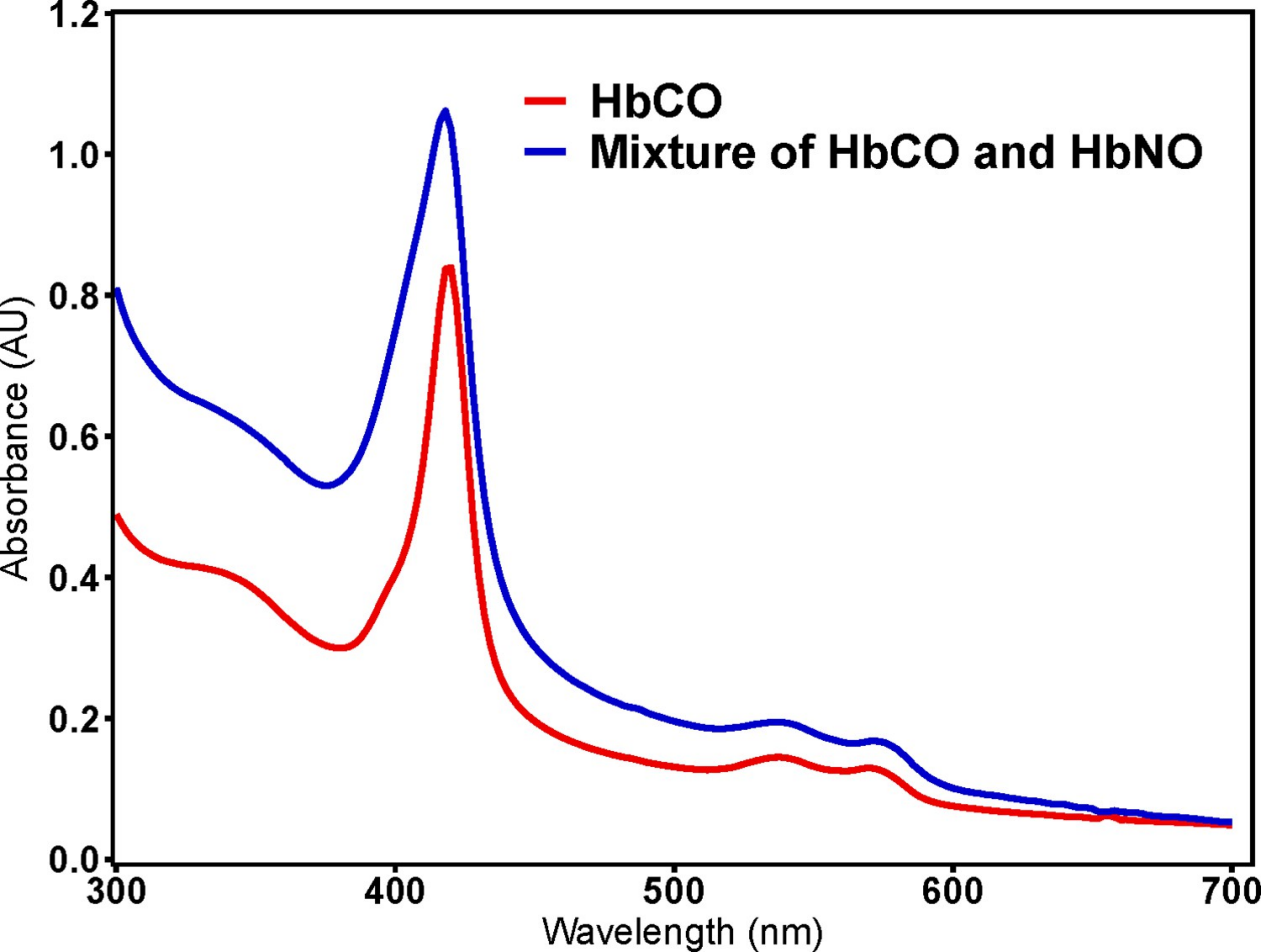
UV-visible spectroscopy of lysed PEG-LEH nanoparticles. A) Soret and (B) Q Bands of NO and CO bound cell free-Hb derived from lysed PEG-LEH nanoparticles encapsulating HbCO (red) and a mixture of HbCO and HbNO (blue).

**Fig 8** compares the UV-visible absorption spectra of lysed PEG-LEH nanoparticles encapsulating HbCO (red) and a mixture of HbCO and HbNO (blue). The spectra obtained after lysing the PEG-LEH nanoparticles encapsulating HbCO had a Soret peak at 419 nm and Q bands at 540 nm and 569 nm (**Fig 8, red**). This observation confirmed that the Hb encapsulated in the PEG-LEH nanoparticles was in the HbCO form. However, the UV-spectra obtained after lysing these vesicles following treatment with ultra-pure NO gas were deemed to be a mixture of HbCO and HbNO (**Fig 8, blue**). These spectra neither corresponded to pure HbCO nor to pure HbNO spectra. Characteristic of pure HbNO, a maximum at 418 nm in the Soret region was observed and the valley between the two Q bands in the visible region was not as pronounced as that of HbCO. The two Q bands, though right-shifted, did not correspond to the Q band maxima for pure HbNO (540 nm and 572 nm as opposed to 545 nm and 575 nm observed for pure HbNO) [52]. We hypothesize that a complete conversion of HbCO to HbNO in PEG-LEH nanoparticles was thwarted by the trans-membrane and the intra-cellular diffusion barriers, which prevented penetration of NO into the core of the PEG-LEH nanoparticles. Spectral deconvolution techniques may reveal individual percentages of these species encapsulated within PEG-LEH nanoparticles.

## Conclusions

Rameez et al [15]. developed a simple method to produce PEG-LEH nanoparticles based on high pressure membrane extrusion. In this study, we produced PEG-LEH nanoparticles using a scalable high-pressure cell disruptor system and performed thorough *in vitro* characterization of their biophysical properties. Our results demonstrate that using the high-pressure cell disruptor methodology developed by our group, we were able to produce consistently large-scale batches of PEG-LEH nanoparticles with high Hb encapsulation. Furthermore, extensive *in vitro* biophysical characterization of these nanoparticles establishes their potential efficacy as viable RBC substitutes.

## Supporting information

S1 Dataset (XLSX)
